# Inhibition of Caspases Improves Non-Viral T Cell Receptor Editing

**DOI:** 10.3390/cancers12092603

**Published:** 2020-09-11

**Authors:** Chunxi Wang, Chun-Chi Chang, Liangli Wang, Fan Yuan

**Affiliations:** Department of Biomedical Engineering, Duke University, Durham, NC 27708, USA; Chunxi.wang@duke.edu (C.W.); chunchi.chang@duke.edu (C.-C.C.); or liangliwng@gmail.com (L.W.)

**Keywords:** CAR-T, electrotransfer, caspase-3, apoptosis, cell viability

## Abstract

**Simple Summary:**

Chimeric antigen receptor (CAR) T cells have shown promising results in cancer treatment. They can be generated with T cells from patients for personalized therapy or donors for off-the-shelf products. A critical step in producing the allogeneic CAR T cells is to knock out the endogenous T cell receptor (TCR). The objective of this study is to determine if inhibition of caspase activities in T cells could increase the cell viability after the TCR knockout mediated by the electrotransfer technology. We observed that inhibition of caspases, especially caspase 3, could significantly improve the cell viability and electrotransfer efficiency. Treatment of cells with caspase inhibitors post electrotransfer could also improve the efficiency of TCR knockout in primary human T cells using electrotransferred ribonucleoprotein. Our data suggest that inhibition of caspases is a promising strategy for improving CAR T cell production.

**Abstract:**

T cell receptor (TCR) knockout is a critical step in producing universal chimeric antigen receptor T cells for cancer immunotherapy. A promising approach to achieving the knockout is to deliver the CRISPR/Cas9 system into cells using electrotransfer technology. However, clinical applications of the technology are currently limited by the low cell viability. In this study, we attempt to solve the problem by screening small molecule drugs with an immortalized human T cell line, Jurkat clone E6-1, for inhibition of apoptosis. The study identifies a few caspase inhibitors that could be used to simultaneously enhance the cell viability and the efficiency of plasmid DNA electrotransfer. Additionally, we show that the enhancement could be achieved through knockdown of caspase 3 expression in siRNA treated cells, suggesting that the cell death in electrotransfer experiments was caused mainly by caspase 3-dependent apoptosis. Finally, we investigated if the caspase inhibitors could improve TCR gene-editing with electrotransferred ribonucleoprotein, a complex of Cas9 protein and a T cell receptor-α constant (TRAC)-targeting single guide RNA (sgRNA). Our data showed that inhibition of caspases post electrotransfer could significantly increase cell viability without compromising the TCR disruption efficiency. These new findings can be used to improve non-viral T cell engineering.

## 1. Introduction

Chimeric antigen receptor (CAR) T cell therapy has been approved to treat patients with various B cell-related tumors, including B-cell precursor acute lymphoblastic leukemia (ALL), diffuse large B-cell lymphoma (DLBCL), primary mediastinal B-cell lymphoma (PMBCL), and high-grade B-cell lymphoma [1]. It relies on the genetic engineering of T cells to express CAR for targeting a specific protein on tumor cell membranes [2,3]. CAR T cells can be generated from the patient’s own T cells or those from healthy donors. The autologous CAR T cells are patient-specific, but it is a challenge to produce them for a subpopulation of cancer patients, and the production platform is currently inefficient for large-scale clinical applications [4]. To avoid these problems, multiplexed genome editing strategies have been used to knock out certain endogenous genes, such as αβ T-cell receptor (TCR), in donor T cells to generate allogeneic universal CAR T cells [5,6], which can be produced massively and applied to treat a large number of patients [5]. The elimination of the αβ TCR is critical for avoiding the graft-versus-host-disease (GVHD) risk in cancer patients [7]. Previous studies have shown that the elimination can happen in cells with T cell receptor-α constant (TRAC) mutation or be achieved through TRAC knockout [6,8]. One of the promising approaches to gene knockout is to deliver the CRISPR/Cas9 system into cells using electrotransfer technology [9].

The technology has been used for the delivery of various molecular cargo into cells, such as DNA, RNA, protein, and ribonucleoprotein (RNP) [10]. It can be applied to all cell types and has few restrictions on the type of molecular cargo being delivered [10]. Moreover, electrotransfer is easy to operate and can be readily scaled up for producing a large amount of cells needed for cell therapy in the clinic [10]. Recently, electrotransfer has been employed in the production of CAR T cells in a clinical trial [11]. Despite these advantages, electrotransfer may result in severe cell death [12,13]. The viability of human primary T cells in gene electrotransfer experiments is highly dependent on the donors, varying from 20% to 40% under optimized experimental conditions for achieving adequate electrotransfer efficiency (e.g., 40%) [13,14,15]. The low viability is a major issue that needs to be tackled before the technology can be applied successfully to manufacturing CAR-T cells. In this study, we hypothesized that inhibition of apoptosis post electrotransfer could increase cell viability. To test the hypothesis, we used both Jurkat clone E6-1, an immortalized human T cell line, and human primary T cells to screen pharmacological inhibitors of apoptosis. We observed that the inhibition of apoptosis could significantly increase both the cell viability and electrotransfer efficiency. Similar results were also observed in experiments using NIH/3T3 cells. Furthermore, we observed that the inhibition of apoptosis had little effects on the TCR disruption efficiency, suggesting that the treatment can be used to improve T cell engineering.

## 2. Results

### 2.1. Treatment of Jurkat Cells with a Pan-Caspase Inhibitor Increases Cell Viability and Electrotransfer Efficiency

**Figure 1:**
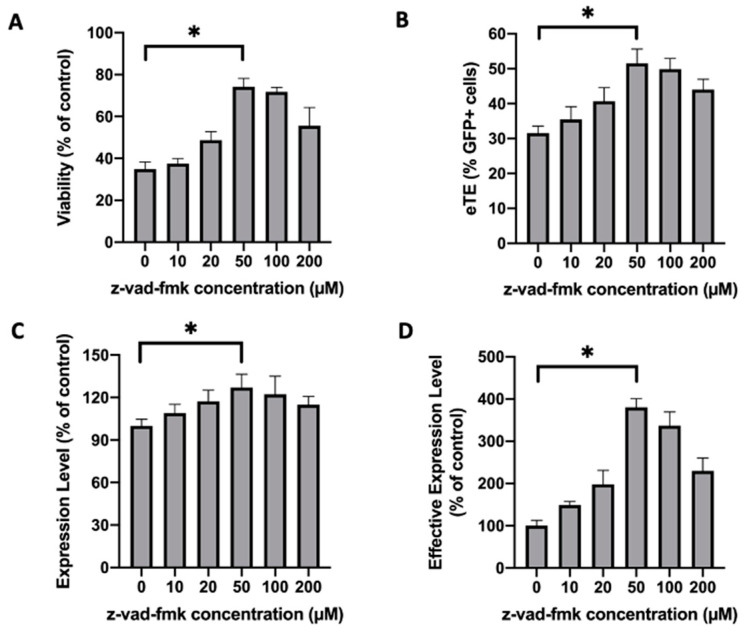
Effects of caspase inhibitor on cell viability and enhanced green fluorescence protein (EGFP) expression in Jurkat cells. Cells were treated with z-vad-fmk at different concentrations for 24 h post pulsing. (**A**) Cell viability is defined as the ratio of propidium iodide (PI) negative cells between the experimental and the non-pulsed control groups. The electrotransfer efficiency was characterized by three parameters: (**B**) Electrotransfer effectiveness (eTE), the percent of PI-cells that expressed the transgene; (**C**) expression level, the geometry mean of transgene expression level among the cells that were PI- and expressed the gene; (**D**) effective expression level, the product of eTE, expression level, and cell viability, which was a measure of the average gene expression level among all cells in a group. Pulsing condition: 650 V/0.2 cm, 400 µs, 1 pulse. Error bars, SEM; * *p* < 0.05, Student’s *t*-test. N (biological replicate number) = 6.

We screened inhibitors of potential molecular targets involved in the apoptosis of Jurkat cells. Examples of the inhibitors tested include a pan-caspase inhibitor (z-vad-fmk), an Ataxia telangiectasia mutated kinase (ATM) inhibitor (KU55933), an ATR inhibitor (VE-821), a cGAS inhibitor (Aspirin), an actin filament inhibitor (cytochalasin B), an apoptosome inhibitor (Apoptosis Inhibitor II), and a calcium chelator (BAPTA-AM). The drug concentration and treatment duration were selected based on the protocols in previous studies [16,17,18,19,20,21]. Through the screening, we identified z-vad-fmk to be the most potent inhibitor of cell death induced by electrotransfer (Figure S1). By incubating Jurkat cells in full culture medium containing z-vad-fmk post electrotransfer, we were able to increase not only the cell viability, but also the electrotransfer efficiency characterized by three parameters: (i) electrotransfer effectiveness (eTE), (ii) expression level, and (iii) effective expression level (see Figure 1). The optimal concentration of z-vad-fmk was found to be 50 µM (Figure 1A–C), at which the cell viability was increased from 35% to 74%, the eTE was increased from 32% to 52%, and the effective expression level was improved by more than 3.8 folds (Figure 1D), compared to the untreated controls. KU55933 could also increase the effective expression level, but the increase came from its ability to improve the eTE instead of the cell viability (Figure S2).

**Figure 2:**
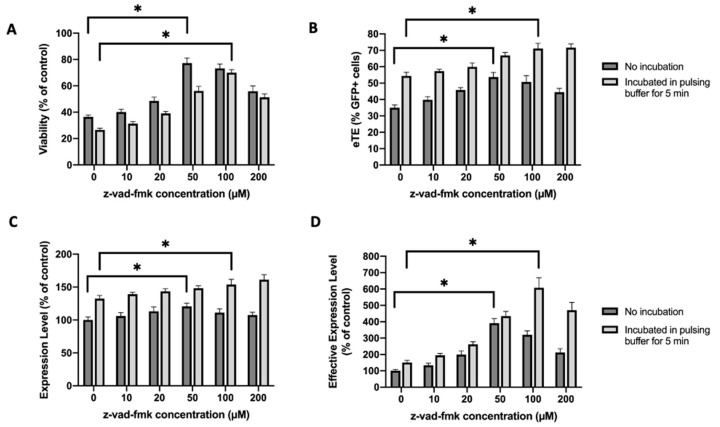
Effects of caspase inhibitor on cell viability and EGFP expression in Jurkat cells under two different experimental conditions. One (i.e., no incubation) was the same as that in Figure 1; the other was similar to the first one except that the cells were incubated in the pulsing buffer for 5 min before being treated with z-vad-fmk at different concentrations for 24 h. (**A**) Cell viability; (**B**) eTE; (**C**) expression level; (**D**) effective expression level. Pulsing condition: 650 V/0.2 cm, 400 µs, 1 pulse. Error bars, SEM; * *p* < 0.05, Student’s *t*-test. N = 6.

We observed in a separate experiment that the eTE could be increased if the cells were incubated at the room temperature in the pulsing buffer for a short period (e.g., 5 min) post electrotransfer, before being transferred into the full medium, compared with transferring the cells immediately. The increase was presumably due to additional cellular uptake of plasmid DNA (pDNA) during this period through macropinocytosis [22]. However, the incubation also reduced cell viability. To prevent cell death, we supplemented the full medium with the caspase inhibitor z-vad-fmk and cultured the cells for 24 h. Our data showed that the z-vad-fmk treatment at 100 µM could completely block the short period incubation-induced cell death (Figure 2A) without compromising the electrotransfer efficiency (Figure 2B–D). Quantitatively, the treatment in the 5-min incubation group increased the cell viability from 26% to 70% (Figure 2A), the eTE from 54% to 71% (Figure 2B), and the effective expression level by more than 6-folds (Figure 2D), compared to the untreated controls.

### 2.2. Caspase-Dependent Apoptosis Is an Important Mechanism of Electrotransfer-Induced Cell Death

**Figure 3:**
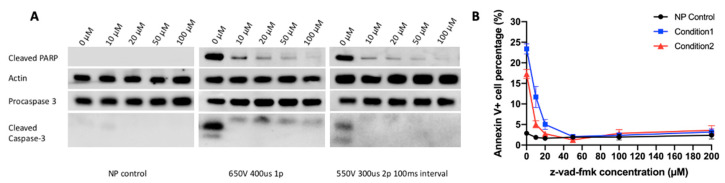
Percent of apoptotic cells after treatment with pharmacological inhibitor. Cells were treated with z-vad-fmk at different concentrations for 8 h post pulsing. (**A**) Western blot analysis at 8 h post pulsing. (**B**) Percent of apoptotic cells (Annexin V+). N = 6. NP, non-pulsed. Two pulsing conditions were tested. Condition 1: 650 V/0.2 cm, 400 µs, 1 pulse; Condition 2: 550 V/0.2 cm, 300 µs, 2 pulses, 10 Hz. Error bars, SEM.

Caspase-dependent apoptosis has been studied after Jurkat cells are treated with nanosecond electric pulses [23,24]. Since pulses with longer durations may activate different cellular pathways [25], we investigated if the electrotransfer with microsecond pulses could cause caspase-dependent cell apoptosis. In the study, we first used Annexin V-based apoptosis assay to examine the percent of apoptotic cells at different time points post electrotransfer in two experimental groups that differed in the pulse sequences used. We observed that the percent of Annexin V positive (Annexin V+) cells was time-dependent (Figure S3), and reached the peak level at approximately 8 h. At this moment, the Western blot analysis showed that the electrotransfer of pDNA caused activation of caspase 3, which in turn cleaved poly(ADP-ribose) polymerase (PARP) (Figure 3A), a downstream substrate of caspase 3. Its cleavage is an indicator of apoptosis.

To block the apoptosis, we treated the pulsed cells with z-vad-fmk for 8 h post electrotransfer, which can simultaneously block functions of multiple caspases, including caspases 1, 3, 7, and 9 (Selleckchem.com). The treatment decreased the percent of Annexin V+ cells in a dose-dependent manner (Figure 3B). The maximal decrease was from 23% to 2% in the first group, and from 17% to 2% in the second group. Since the 2% level was statistically the same as that in the non-pulsed control groups, the data indicated that by inhibiting multiple caspases, we could completely block cell apoptosis induced by electrotransfer.

### 2.3. Caspase 3 Is a Key Mediator in Electrotransfer-Induced Cell Apoptosis

**Figure 4:**
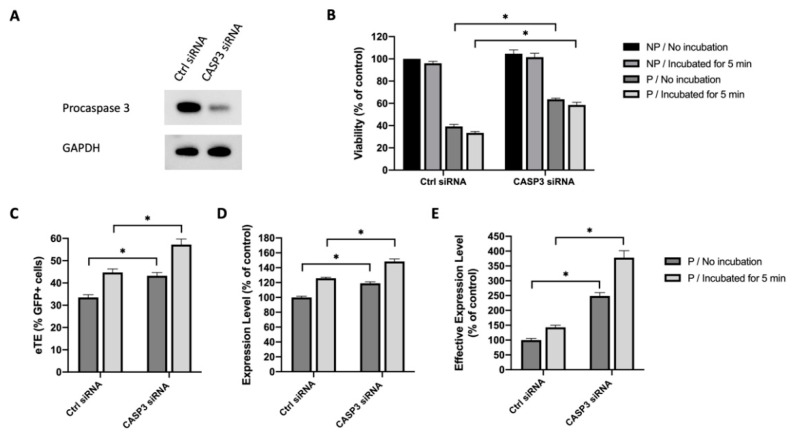
Effects of procaspase 3 knockdown on cell viability and EGFP expression in Jurkat cells. (**A**) Western blot analysis showing the reduction in procaspase 3 expression after the gene knockdown with CASP3 siRNA treatment. (**B**) Cell viability. Cells were either incubated in the pulsing buffer for 5 min, or transferred to full culture medium immediately after pulsing. (**C**) eTE; (**D**) expression level; (**E**) effective expression level. Pulsing condition: 650 V/0.2 cm, 400 µs, 1 pulse. NP, non-pulsed control; P, pulsed cells. Error bars, SEM; * *p* < 0.05, Student’s *t*-test, N = 6.

Caspase 3 is one of the executioner caspases that are activated by proteolytic cleavage during the apoptotic process. Previous studies have shed light on the role of caspase 3 played in controlling cell apoptosis under various conditions [26,27,28]. Thus, we hypothesized that caspase 3 was a key molecule in mediating cell apoptosis caused by electrotransfer, and that inhibiting caspase 3 alone could lead to an increase in cell viability. To test the hypothesis, we used a small interfering RNA (siRNA) to knock down procaspase 3 expression in Jurkat cells. At 48 h after the siRNA or a scrambled control siRNA were delivered into Jurkat cells, the cells were collected either for Western blot analysis to confirm the knockdown of procaspase 3 (Figure 4A), or for electrotransfer experiments. At 24 h post pDNA electrotransfer, we observed that knocking down procaspase 3 could increase cell viability, compared to the matched controls. Quantitatively, the increase was from 39% to 64% if the cells were transferred immediately into the full medium after pulsing, or from 33% to 59% if the cells were incubated in the pulsing buffer at the room temperature for 5 min before being transferred to the medium (Figure 4B). The knockdown of procaspase 3 expression also significantly increased the electrotransfer efficiency characterized by the eTE, the expression level, and the effective expression level (Figure 4C–E).

### 2.4. Similar Effects of Caspase Inhibition Are Observed in NIH/3T3 Fibroblasts

**Figure 5:**
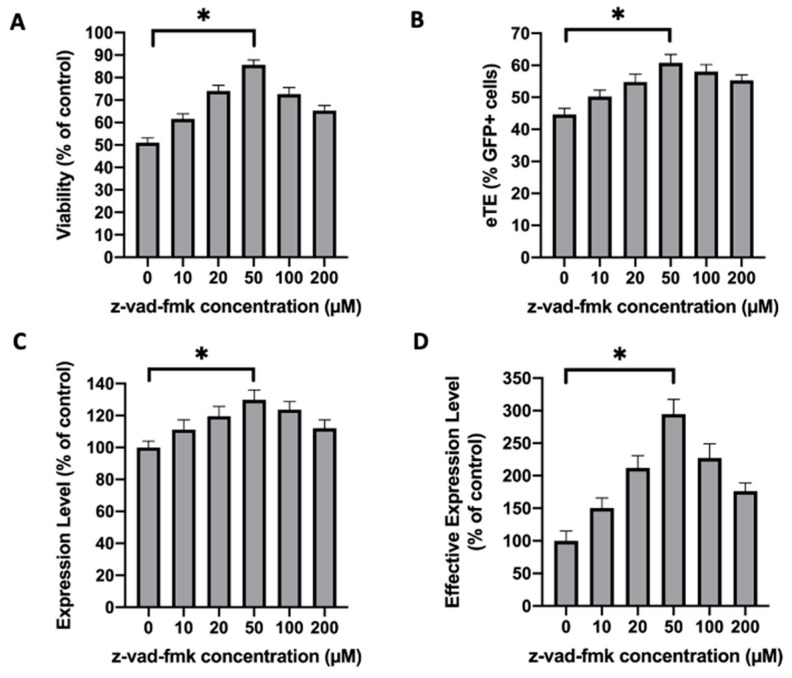
Effects of caspase inhibitor on cell viability and EGFP expression in NIH/3T3 cells. The experimental condition was similar to that in Figure 1, except that NIH/3T3 instead of Jurkat cells were used in the experiment. (**A**) Cell viability; (**B**) eTE; (**C**) expression level; (**D**) effective expression level. Pulsing condition: 650 V/0.2 cm, 400 µs, 1 pulse. Error bars, SEM; * *p* < 0.05, Student’s *t*-test. N = 6.

**Figure 6:**
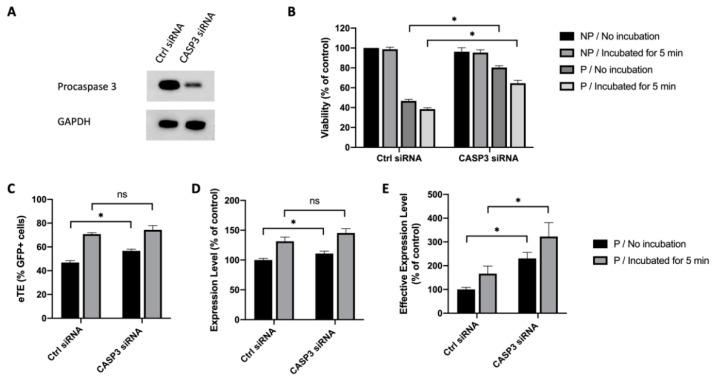
Effects of procaspase 3 knockdown on cell viability and EGFP expression in NIH/3T3 cells. (**A**) Western blot analysis showing the reduction in procaspase 3 expression after procaspase 3 knockdown with CASP3 siRNA treatment; (**B**) Cell viability; (**C**) eTE; (**D**) expression level; (**E**) effective expression level. Pulsing condition: 650 V/0.2 cm, 400 µs, 1 pulse. NP, non-pulsed control; P, pulsed cells; ns, non-significant. Error bars, SEM; * *p* < 0.05, Student’s *t*-test, N = 6.

To confirm the data obtained with Jurkat cells, we repeated the experiments with another cell line, NIH/3T3. Unlike the Jurkat cells, which need to grow in suspension, NIH/3T3 is a mouse embryonic fibroblast and has to be cultured as monolayers. In the study, we treated the NIH/3T3 cells with z-vad-fmk at different concentrations (0–200 µM) for 24 h post pDNA electrotransfer, and measured the cell viability and electrotransfer efficiency at the end of the treatment. At the optimal concentration, which was observed to be 50 µM, the treatment increased the cell viability from 51% to 86% (Figure 5A), the eTE from 45% to 61% (Figure 5B), the expression level from 100% to 130% (Figure 5C), and the effective expression level by 2.9 folds (Figure 5D). To examine whether caspase 3 played a key role in electrotransfer-induced death of NIH/3T3 cells, we knocked down the procaspase 3 expression using the siRNA. At 48 h post siRNA treatment, the cells were divided into two groups for confirming the knockdown of procaspase 3 expression with Western blot analysis (Figure 6A), and electrotransfer experiment, respectively. At 24 h post electrotransfer, we observed that the knockdown of procaspase 3 expression significantly increased the cell viability and electrotransfer efficiency (Figure 6B–E), which were consistent with the observations in experiments using Jurkat cells.

### 2.5. Inhibition of Apoptosis Improves Gene Editing Efficiency

**Figure 7:**
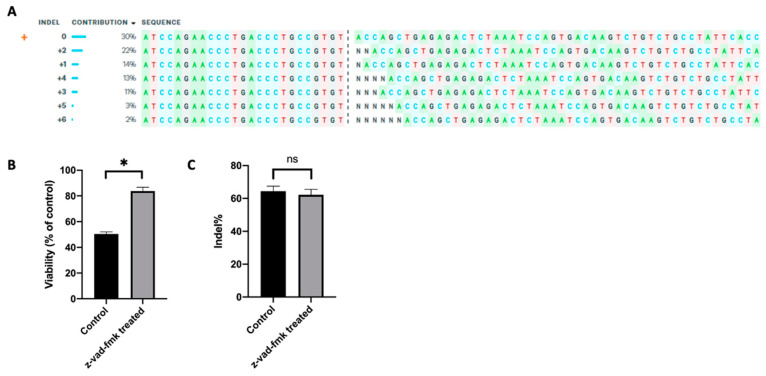
Effects of apoptosis inhibition on cell viability and indel frequency in Jurkat cells. The gene-editing was achieved through electrotransfer of a ribonucleoprotein (RNP) targeting the T cell receptor-α constant (TRAC) gene. (**A**) Inference of CRISPR Edits (ICE) analysis, showing the outcome of TRAC editing in Jurkat cells; (**B**) cell viability measured at 24 h; (**C**) indel within the TRAC gene determined at 48 h post electrotransfer using TIDE analysis. Pulsing condition: 650 V/0.2 cm, 300 µs, 2 pulses, 10 Hz. ns, non-significant. Error bars, SEM; * *p* < 0.05, Student’s *t*-test, N = 6.

The new strategy described above was applied to improving gene-editing efficiency. As mentioned in the Introduction section, an important step in the production of universal CAR T cells is to eliminate the αβ TCR for avoiding GVHD risk in cancer patients [7]; and the elimination can be achieved through TRAC knockout [6,8]. To show that inhibition of apoptosis could improve the outcome of gene knockout, we electrotransferred an RNP, a complex of Cas9 protein and TRAC-targeting sgRNA, into Jurkat cells. After the electrotransfer, the cells were cultured either in the medium supplemented with 50 µM z-vad-fmk or in the control medium with an equal amount of DMSO. The outcome of TRAC editing in Jurkat cells was confirmed using Inference of CRISPR Edits (ICE) analysis (Figure 7A), and the frequency of insertion or deletion (indel) within the TRAC gene was determined using the TIDE analysis. At 24 h post electrotransfer, we observed that treating the cells with z-vad-fmk increased the cell viability from 50% to 84%, compared to the untreated control (Figure 7B), demonstrating that inhibiting caspases could reduce cell loss induced by RNP electrotransfer. The data was consistent with those described above, where the cell loss was caused by pDNA electrotransfer. However, the inhibition of caspases led to an insignificant change in the indel quantified at 48 h post electrotransfer (Figure 7C). Since the viability was increased and the efficiency was unchanged, the data indicated that the total number of viable cells with the TRAC gene knocked out was increased.

### 2.6. Inhibition of Caspase 3 in Human Primary T Cells Improves Gene Editing Efficiency

**Figure 8:**
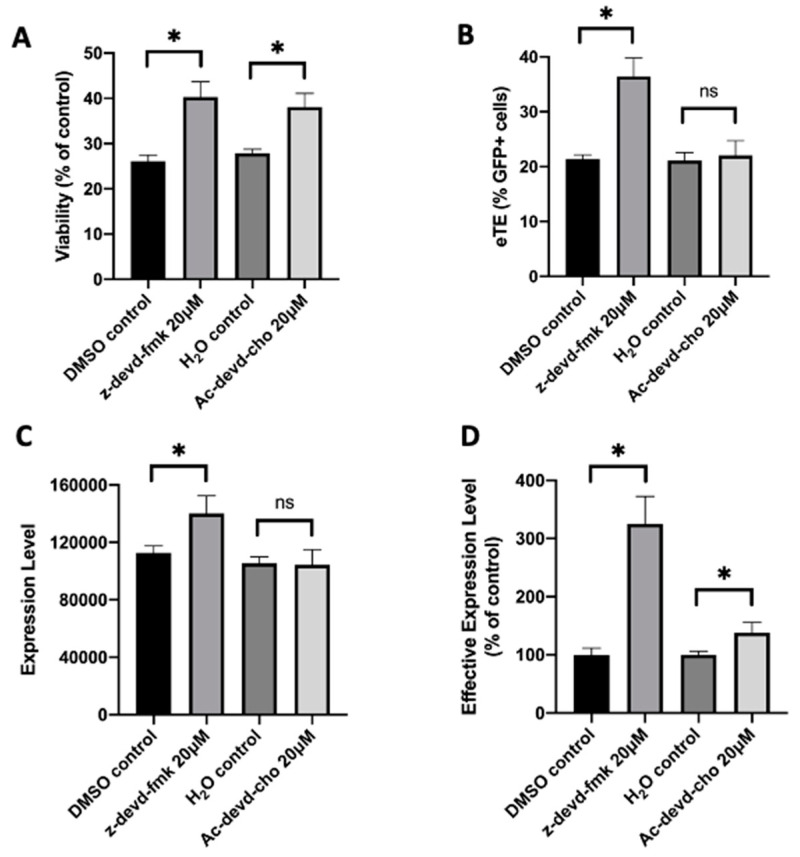
Effects of caspase inhibitors on cell viability and EGFP expression in human primary T cells. The experimental condition was similar to that in Figure 1, except that human primary T cells instead of Jurkat cells were used in the experiment, and that z-devd-fmk and Ac-devd-cho, instead of z-vad-fmk, were used to inhibit cell apoptosis. (**A**) Cell viability; (**B**) eTE; (**C**) expression level; (**D**) effective expression level. Pulsing condition: 650 V/0.2 cm, 400 µs, 1 pulse. ns, non-significant. Error bars, SEM; * *p* < 0.05, Student’s *t*-test. N = 4.

To further demonstrate the capability of caspase inhibition for improving CAR T cell production, we treated human primary T cells with z-vad-fmk after pDNA or RNP electrotransfer. Our data showed that although the compound was nontoxic to T cells without electrotransfer, the treatment was highly toxic. At the concentrations that worked for Jurkat cells, the treatment with z-vad-fmk killed the majority of human T cells (Figure S4A,B). Even at 0.5 µM, which was 100 times lower than the optimal concentration for Jurkat cells, the inhibitor treatment still killed approximately half of the cell population, compared to the untreated controls (Figure S4C–F). To reduce the toxicity caused presumably by non-specific inhibition of all caspases, we tested inhibitors more specific to caspase 3, such as z-devd-fmk and Ac-devd-cho. We observed that both inhibitors could increase the T cell viability by ~30% at the optimal treatment concentrations, compared to the matched controls (Figure 8A, Figure S5A). Similar to the effects of procaspase 3 siRNA treatment on Jurkat cells described above, inhibition of caspase 3 in human T cells with z-devd-fmk treatment at 20 µM could effectively enhance the electrotransfer efficiency (Figure 8B–D, Figure S5B–D). The treatment of T cells with Ac-devd-cho resulted in insignificant or minor changes in the electrotransfer efficiency (Figure 8B–D, Figure S5B–D).

**Figure 9:**
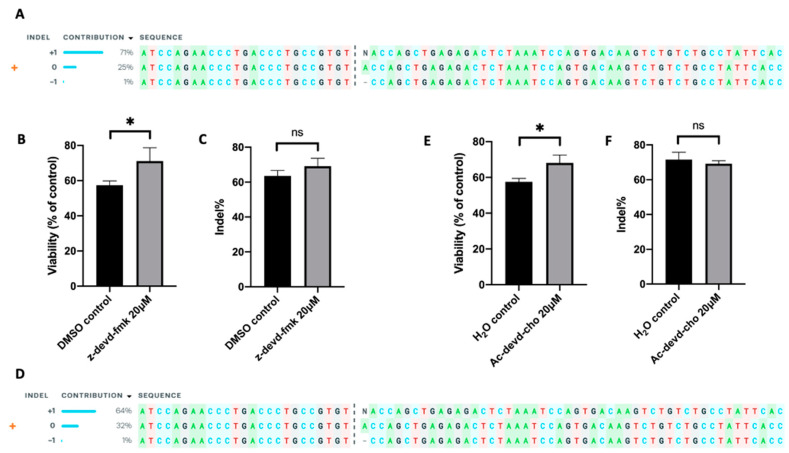
Effects of apoptosis inhibition on cell viability and indel frequency in human primary T cells. The gene-editing was achieved through electrotransfer of an RNP targeting the TRAC gene. (**A**–**C**) Apoptosis was inhibited with z-devd-fmk treatment; (**D**–**F**) apoptosis was inhibited with Ac-devd-cho treatment. (**A**,**D**) ICE analysis, showing the outcome of TRAC editing in T cells; (**B**,**E**) cell viability measured at 24 h post pulsing; (**C**,**F**) Indel within TRAC gene determined at 48 h post pulsing using TIDE analysis. Pulsing condition: 650 V/0.2 cm, 300 µs, 2 pulses, 10 Hz. ns, non-significant. Error bars, SEM; * *p* < 0.05, Student’s *t*-test, N = 4.

Further, we tested the effect of caspase 3 inhibition on improving TRAC knockout efficiency in human primary T cells. Similar to those for Jurkat cells described above, we electrotransferred an RNP into human T cells, followed by the treatment of the cells with either inhibitor (z-devd-fmk or Ac-devd-cho) at 20 µM or equal amount of solvent (DMSO or H_2_O). The TRAC editing in T cells was confirmed using ICE analysis (Figure 9A,D), and the indel was determined using the TIDE analysis. At 24 h post electrotransfer, the inhibition of caspase 3 with z-devd-fmk or Ac-devd-cho treatment increased T cell viability from 57% to 71%, or from 57% to 68%, respectively, compared to the matched controls (Figure 9B,E). The same treatments insignificantly alter the indel quantified at 48 h post electrotransfer (Figure 9C,F). These data demonstrated that inhibition of caspase 3 could effectively increase the total number of gene-edited human T cells by means of increasing cell viability without decreasing the percent of gene-edited cells.

## 3. Discussion

Data in the study showed that electrotransfer-induced cell death was primarily caused by caspase 3-dependent apoptosis. It could be significantly reduced through the treatment of cells with pharmacological inhibitors of apoptosis post electrotransfer or knockdown of procaspase 3 expression prior to electrotransfer. Concomitantly, inhibition of apoptosis could improve the electrotransfer efficiency in terms of the percent of cells expressing transgenes, the level of transgene expression, and the gene-editing efficiency.

Apoptosis networks are complicated and involve many players [29]. Mechanisms of cell death induced by nanosecond electric pulses have been previously investigated with various cell lines, including Jurkat cells [23,24]. However, few studies in the literature have focused on cell death caused by electric pulses with a longer duration. In a recent study, Bosnjak et al. showed that gene electrotransfer with millisecond pulses could cause an inflammatory response and lead to growth delay in melanoma through activating cytosolic DNA sensors and caspase-dependent apoptotic pathways [30]. In the current study, we observed that caspases, especially caspase 3, played an important role in cell death induced by electrotransfer with sub-millisecond pulses, and that knocking down its expression could greatly enhance the outcome of T cell engineering.

However, we cannot rule out that other molecules in apoptosis-related or unrelated pathways can also function as mediators of cell death caused by electrotransfer. In fact, our data show that inhibiting caspase activities cannot bring the cell viability to the same level as that in the non-pulsed control groups, indicating that the inhibition of apoptosis alone can reduce cell death, but is insufficient to block it. More effort is needed to thoroughly understand the regulatory networks involved in cell death that can be affected by electrotransfer.

The current study also showed that the apoptosis could be caused by electrotransfer of molecular cargo or electric pulsing alone. The data are consistent with the observations in the literature. Exposure of cells to strong electric pulses can cause severe plasma membrane damage, such as permeabilization and oxidation [31]. The permeabilization allows the influx of calcium and other small molecules to trigger apoptosis [32]. Lipid peroxidation and generation of reactive oxygen species (ROS) in cells can lead to oxidative stress and cell death [33]. Further, electrotransfer of DNA or RNA into cells may enlarge the areas of the plasma membrane injured initially by electric pulsing, causing additional damage to the membrane. Meanwhile, the internalized DNA or RNA cargo can be recognized by cytosolic sensors in the innate immune system commonly used for the detection of invaded pathogens [34,35]. The recognition initiates immune responses, which may then activate multiple cell death pathways. Interestingly, our data suggested that the activation of apoptosis triggered by either the membrane damage or the cargo internalization shares the same pathways that could be inhibited by caspase inhibitor treatment or procaspase 3 knockdown.

## 4. Materials and Methods

### 4.1. Cell Culture

Jurkat Clone E6-1 (ATCC, Manassas, VA, USA) cells were cultured in Corning^®^ T-75 flasks in RPMI 1640 medium (Thermo Fisher Scientific, Waltham, MA, USA) supplemented with 10% bovine calf serum (BCS, Avantor Seradigm, Radnor Township, PA, USA) and 1% Pen-Strep (P/S, Thermo Fisher Scientific). Cell density was maintained at 1–2 × 10^6^ cells/mL. NIH/3T3 (ATCC) cells were cultured on 10 cm tissue culture dishes in DMEM supplemented with 10% BCS and 1% P/S. Cell density was maintained at 75–90% confluency. Human primary T cells were isolated from peripheral blood mononuclear cells (Zenbio, Research Triangle Park, NC, USA), and stimulated with CD3/CD28 dynabeads (Thermo Fisher Scientific) for 3 days before experiments. They were culture in AIM-V medium (Thermo Fisher Scientific) with 10% human serum (Geimini Bio, Calabasas, CA, USA), 1% P/S, and 200 unit/mL human IL-2 (Miltenyi Biotec, Bergisch Gladbach, Germany). All cells were maintained at 37 °C in a humidified incubator with 5% CO_2_ and passaged every 1–2 days. All cell lines used in the study were tested negative for mycoplasma.

### 4.2. Electrotransfer of pDNA

Before electrotransfer, Jurkat or T cells in suspension were harvested by centrifugation, and the cell pellet was re-suspended in 100 μL electric pulsing buffer (5 mM KCl, 30 mM MgCl_2_, 120 mM Na_2_HPO4/NaH_2_PO4 (pH = 7.4), 50 mM Mannitol [36]) at a concentration of 10^7^ cells/mL. NIH/3T3 cells were detached from the culture dish surface using 0.25% trypsin-EDTA (Thermo Fisher Scientific), harvested by centrifugation, and then re-suspended in 100 μL pulsing buffer at a concentration of 10^7^ cells/mL. After adding 1 μg pDNA (pEGFP-N1, Nova Lifetech lnc, Hong Kong, China), the cell suspension was transferred to a disposable 2-mm gap aluminum cuvettes (Bio-Rad, Hercules, CA, USA), and pulsed by using the BTX ECM 830 Square Wave Electroporation System (Harvard Apparatus, Holliston, MA, USA). Pulsing conditions for different cell lines are indicated in the Results or Supplementary Information section.

### 4.3. Treatment of Cells with Apoptosis Inhibitors

The inhibitors, z-vad-fmk, z-devd-fmk, and Ac-devd-cho, were purchased from Selleckchem (Houston, TX, USA; Catalog No. S7023, S7312, S7901). z-vad-fmk and z-devd-fmk were first dissolved in DMSO and then diluted in culture medium to achieve the final concentrations, as indicated in figure legends. Ac-devd-cho was first dissolved in ddH_2_O and then diluted with culture medium to achieve the final concentrations, as indicated in figure legends. Cells were either transferred to the diluted solution immediately post electrotransfer, or kept in the pulsing buffer at room temperature for 5 min, then transferred to the solution. Thereafter, the cells were cultured normally for 24 h before being analyzed.

### 4.4. siRNA Knockdown of Procaspase-3 Expression

For knocking down procaspase-3 expression in Jurkat cells and NIH/3T3 fibroblasts, either human or mouse caspase-3 siRNA (Santa Cruz Biotechnology, Dallas, TX, USA) was electrotransferred (650 V/0.2 cm, 400 µs, 1 pulse) into cells by using the same protocol as that for pDNA delivery described above [37]. After electrotransfer, the cells were cultured in full medium for 48 h. Then, cells were collected and analyzed by Western blot to determine the knockdown efficiency, or used for pDNA electrotransfer experiments.

### 4.5. Western Blot Analysis

All cell samples were collected at either 8 h post-DNA electrotransfer or 48 h post-siRNA electrotransfer. The analysis was performed based on a protocol described in our previous study [38]. Briefly, proteins were extracted from cell lysates through centrifugation. The extract was resolved on a NuPAGE^TM^ 4–12% Bis-Tris gel (Thermo Fisher Scientific) and then transferred onto the nitrocellulose membrane. The membrane was blocked by incubating with 5% blotting-grade blocker (Bio-Rad) in PBST (PBS + 0.05% Tween 20) at room temperature for 30 min. After blocking, the membrane was incubated with the primary antibodies (1:1000 dilution) at 4 °C overnight. On the second day, the membrane was washed and incubated with horseradish peroxidase (HRP)-linked anti-rabbit IgG (1:1000 dilution, GE Healthcare, Chicago, IL, USA) for 1 h. After the incubation, the membrane was washed again before imaging. To enhance the signal in Western blot analysis, SuperSignal™ West Femto Maximum Sensitivity Substrate (Thermo Fisher Scientific) was used following the manufacturer’s protocol. Excess reagent was removed from the membrane; and chemiluminescence signals were detected using a ChemiDoc^TM^ Touch imaging system (Bio-Rad). The following primary antibodies were used in the study: (i) Apoptosis Western blot cocktail for detection of pro/p17-caspase-3, cleaved PARP1, and muscle actin (Abcam, Cambridge, UK); (ii) recombinant anti-pro caspase-3 antibody [E61] (Abcam) and recombinant anti-pro caspase-3 antibody [E83-103] (Abcam) for detection of procaspase-3 in Jurkat and NIH/3T3 cells; and (iii) GAPDH Rabbit mAb (Cell Signaling Technology, Danvers, MA, USA) for detection of the endogenous level of total GAPDH protein in Jurkat and NIH/3T3 cells. The original images of Western blot can be found in Supplementary Materials (Figures S6–S9).

### 4.6. Flow Cytometry Analysis

Flow cytometry analysis was performed to quantify cell viability, electrotransfer efficiency, and apoptosis status [22,38]. In the apoptosis assay, cells in experimental groups and matched controls were collected at 8 h post-electrotransfer of pDNA. Cells in each sample were washed twice at room temperature in PBS, and re-suspended in annexin-binding buffer (10 mM HEPES, 140 mM NaCl, and 2.5 mM CaCl_2_, pH 7.4) at a density of ~1 × 10^6^ cells/mL. 100 μL of the cell suspension was gently mixed with 5 μL of Annexin V Alexa Fluor™ 594 conjugate (Thermo Fisher Scientific), incubated at room temperature for 15 min, then diluted gently with 400 μL of the annexin-binding buffer. The sample was kept on ice before being analyzed with flow cytometry. In cell viability and electrotransfer efficiency assays, cell samples were collected 24 h post-electrotransfer, stained with 5 μg/mL PI, and analyzed with flow cytometry. A flow cytometry analysis was performed using a BD FACSCanto II flow cytometer (Becton Dickinson, Franklin Lakes, NJ, USA). All flow cytometry data were analyzed using FlowJo (BD, Ashland, OR, USA).

### 4.7. TRAC Knockout by Cas9/sgRNA RNP in Jurkat Cells

spCas9 protein (Millipore Sigma, St. Louis, MO, USA) and TRAC sgRNA (AGAGTCTCTCAGCTGGTACA; IDT, San Jose, CA, USA) were mixed at the final concentrations of with 150 pmol and 300 pmol, respectively, incubated at room temperature for 30 min to form RNP, and then electrotransferred into cell samples, using the protocol described above. Thereafter, the cells were cultured in full medium supplemented with z-vad-fmk at 50 mM unless otherwise indicated. 24 h post electrotransfer, the cell viability was determined using the flow cytometry; and the genomic DNA was extracted. The targeted locus was amplified with PCR (Forward primer: TTGCTGGGGTTTTGAAGAAG; Reverse Primer: GGTTTTGGTGGCAATGGATA) for DNA sequence analysis (Genewiz, South Plainfield, NJ, USA; sequencing primer: TTGTCCATCACTGGCATCTG). The TRAC editing in T cells was confirmed with the ICE analysis [39]; and the gene-editing efficiency was determined using the TIDE analysis [40].

## 5. Conclusions

The electrotransfer of molecular cargo into cells can induce apoptosis. Data in the current study demonstrate that apoptosis can be inhibited by the treatment of cells with small molecule inhibitors of caspases post electrotransfer or knockdown of procaspase 3 expression prior to electrotransfer. Furthermore, our data show that specific inhibitors for caspase 3, such as z-devd-fmk and Ac-devd-cho, are significantly less toxic to T cells than pan-caspase inhibitors. Thus, they can be used to improve the efficiency of gene-editing in human primary T cells. Results from the study suggest that inhibition of caspases is a promising strategy for improving CAR T cell production, and more generally, electrotransfer of molecular cargo in cell engineering applications.

## Supplementary Materials

The following are available online at https://www.mdpi.com/2072-6694/12/9/2603/s1, Figure S1: Effects of small molecule drugs on cell viability and EGFP expression in Jurkat cells. Cells were either pretreated with cytochalasin B (2 µM), BAPTA-AM (5 µM), Aspirin (1 mM), or apoptosis inhibitor II (50 µM) for 60 min before pulsing, or treated with VE-821 (5 µM), KU55933 (5 µM), or z-vad-fmk (20 µM) for 24 h post-pulsing. The drug concentration and treatment duration were selected based on the protocols in previous studies [16,17,18,19,20,21]. A. Cell viability; B. eTE; C. expression level; D. effective expression level. Pulsing condition: 650 V/0.2 cm, 400 µs, 1 pulse. Error bars, SEM; * *p* < 0.05, Student’s *t*-test. N (biological replicate number) = 4. Figure S2: Effects of KU55933 on cell viability and EGFP expression in Jurkat cells. Cells were treated with KU55933 at different concentrations for 24 h post-pulsing. A. Cell viability; B. eTE; C. expression level; D. effective expression level. Pulsing condition: 650 V/0.2 cm, 400 µs, 1 pulse. Error bars, SEM; * *p* < 0.05, Student’s *t*-test. N = 4. Figure S3: Effects of z-vad-fmk treatment on apoptosis of Jurkat cells. The percent of apoptotic cells was quantified with the Annexin V apoptosis assay. The experimental condition was similar to that in Figure 1, except that the cells were collected at 1, 2, 4, 8, and 24 h post pulsing by centrifugation. The peak of apoptosis occurred at ~8 h. Pulsing condition: 650 V/0.2 cm, 400 µs, 1 pulse. Each symbol represents data from a single experiment. Figure S4: Effects of z-vad-fmk treatment on the viability of human primary T cells. The experimental condition was similar to that in Figure 1, except that human primary T cells instead of Jurkat cells, were used in the experiment. A. Flow cytometry analysis of human T cells at 24 h after pulsing without z-vad-fmk treatment; B. flow cytometry analysis of human T cells at 24 h after pulsing with 2 µM z-vad-fmk treatment. More than 70% of treated T cells became PI positive. C. Cell viability; D. eTE; E. expression level; F. effective expression level for samples treated with z-vad-fmk at different concentrations. Pulsing condition: 650 V/0.2 cm, 400 µs, 1 pulse. N = 3. Figure S5: Effects of two caspase inhibitors (z-devd-fmk and Ac-devd-cho) on cell viability and EGFP expression in human primary T cells. The experimental condition was similar to that in Figure 1, except that human primary T cells instead of Jurkat cells, were used in the experiment, and that z-devd-fmk and Ac-devd-cho, instead of z-vad-fmk, were used to inhibit cell apoptosis. A. Cell viability; B. eTE; C. expression level; D. effective expression level. Pulsing condition: 650 V/0.2 cm, 400 µs, 1 pulse. ns, non-significant. Error bars, SEM; * *p* < 0.05, Student’s *t*-test. N = 3. Figure S6: Original Western blot images used to generate the NP control panel in Figure 3A. Jurkat cells were treated with z-vad-fmk at different concentrations for 8 h post pulsing. Western blot membrane was first imaged as a whole (A), then cut horizontally into three parts (B–D) to achieve optimal exposure time for imaging of different protein bands. A. image of the whole membrane; B. membrane portion containing the cleaved PARP bands; C. membrane portion containing the cleaved caspase 3 bands; D. membrane portion containing the actin bands. Pulsing condition: 650 V/0.2 cm, 400 µs, 1 pulse. Lane 1: 0 μM; Lane 2: 10 μM; Lane 3: 20 μM; Lane 4: 50 μM; Lane 5: 100 μM. Lane 6–10: Repeats of lane 1–5; Lane 11&12: Pulsed samples (positive controls). Figure S7: Original Western blot images used to generate the two panels for pulsed groups in Figure 3A. Jurkat cells were treated with z-vad-fmk at different concentrations for 8 h post pulsing. Western blot membrane was first imaged as a whole (A), then cut horizontally into three parts (B–D) to achieve optimal exposure time for imaging of different protein bands. A. image of the whole membrane; B. membrane portion containing the cleaved PARP bands; C. membrane portion containing the cleaved caspase 3 bands; D. membrane portion containing the actin bands. Pulsing condition for Lane 1–6: 650 V/0.2 cm, 400 µs, 1 pulse. Lane 1: 0 μM; Lane 2: 10 μM; Lane 3: 20 μM; Lane 4: 50 μM; Lane 5: 100 μM. Lane 6: NP control (negative control); Pulsing condition for Lane 7–12: 550 V/0.2 cm, 300 µs, 2 pulses, 1 Hz. Lane 7: 0 μM; Lane 8: 10 μM; Lane 9: 20 μM; Lane 10: 50 μM; Lane 11: 100 μM. Lane 12: NP control (negative control). Figure S8: Original Western blot images used to generate Figure 4A. Jurkat cells were treated with either non-targeting control siRNA (Ctrl siRNA) or procaspase 3 siRNA (CASP3 siRNA). A. image of the whole membrane; B. membrane portion containing the GAPDH bands; C. membrane portion containing the procaspase 3 bands. Lane 1&5: Cells treated with CASP3 siRNA (sample 1); Lane 2&6: Cells treated with Ctrl siRNA (sample 1); Lane 3&7: Cells treated with CASP3 siRNA (sample 2); Lane 4&8: Cells treated with Ctrl siRNA (sample 2). During the primary antibody incubation, lane 1–4 were incubated with procaspase 3 antibody, and lane 5–8 were incubated with GAPDH antibody. The bands of the two samples were similar to each other. Thus, only the bands of sample 1 were reported in Figure 4A. Figure S9: Original Western blot images used to generate Figure 6A. NIH/3T3 cells were treated with either non-targeting control siRNA (Ctrl siRNA) or procaspase 3 siRNA (CASP3 siRNA). A. image of the whole membrane; B. membrane portion containing the GAPDH bands; C. membrane portion containing the procaspase 3 bands. Lane 1&5: Cells treated with Ctrl siRNA (sample 1); Lane 2&6: Cells treated with CASP3 siRNA (sample 1); Lane 3&7: Cells treated with Ctrl siRNA (sample 2); Lane 4&8: Cells treated with CASP3 siRNA (sample 2). During the primary antibody incubation, lane 1–4 were incubated with procaspase 3 antibody, and lane 5–8 were incubated with GAPDH antibody. The bands of the two samples were similar to each other. Thus, only the bands of sample 2 were reported in Figure 6A.
