# Supplementary material for: Inhibition of Caspases Improves Non-Viral T Cell Receptor Editing
Source: Cancers (Basel). 2020 Sep 11;12(9):2603. doi: 10.3390/cancers12092603 (PMC7565551; doi:10.3390/cancers12092603)
Supplement: Supplementary file 1 [file cancers-12-02603-s001.pdf]

Supplementary Material:

Inhibition of Caspases Improves Non-Viral T Cell Receptor Editing

Chunxi Wang, Chun-Chi Chang, Liangli Wang and Fan Yuan

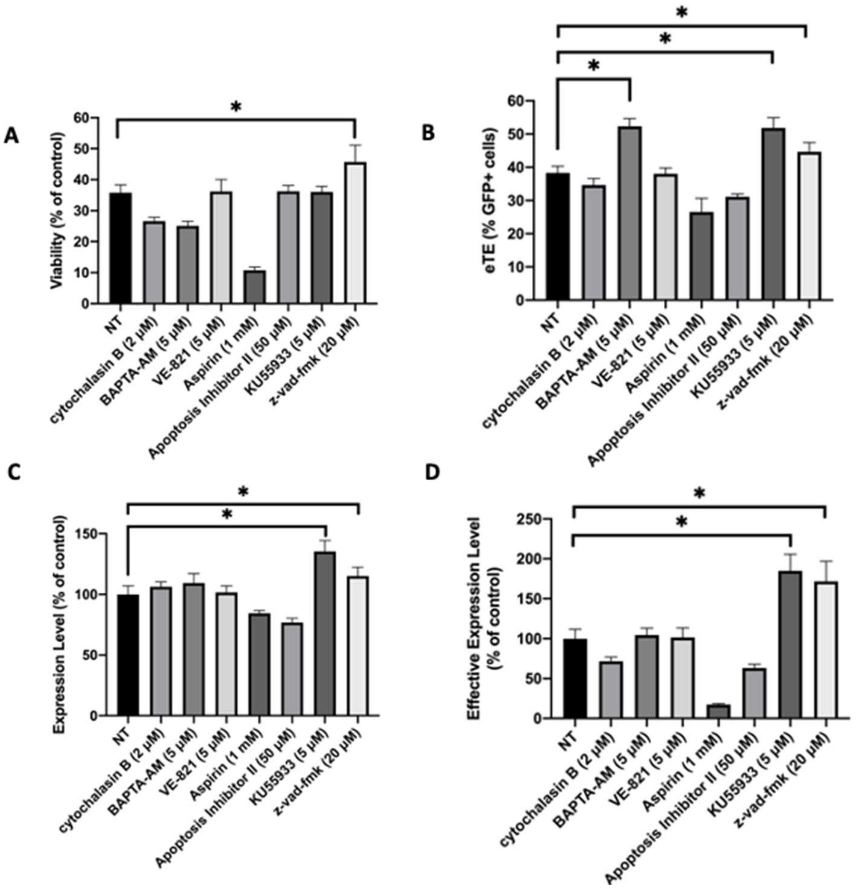

Figure S1. Effects of small molecule drugs on cell viability and EGFP expression in Jurkat cells. Cells were either pretreated with cytochalasin B (2 μM), BAPTA-AM (5 μM), Aspirin (1 mM), or apoptosis inhibitor II (50 μM) for 60 minutes before pulsing, or treated with VE-821 (5 μM), KU55933 (5 μM), or z-vad-fmk (20 μM) for 24 hours post-pulsing. The drug concentration and treatment duration were selected based on the protocols in previous studies [16-21]. **(A)** Cell viability; **(B)** eTE; **(C)** Expression Level; **(D)** Effective Expression Level. Pulsing condition: 650 V/0.2 cm, 400 μs, 1 pulse. Error bars: SEM; * $P < 0.05$, Student's t-test. N (biological replicate number) = 4.

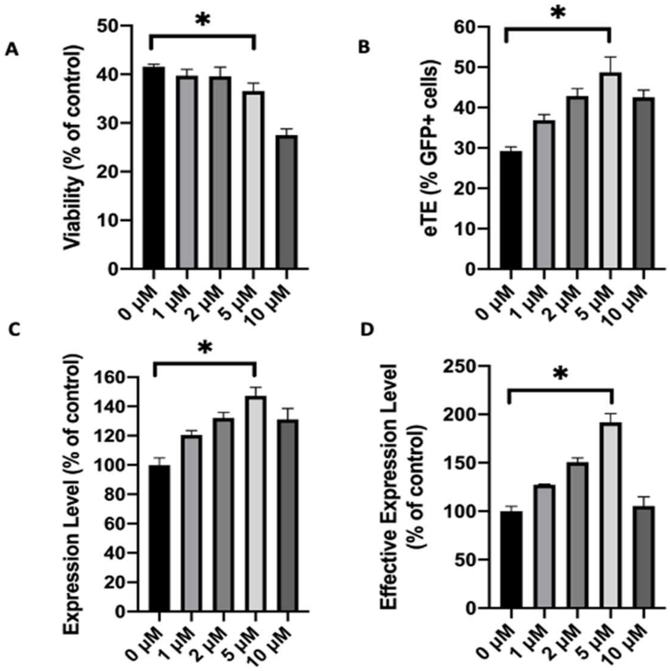

Figure S2. Effects of KU55933 on cell viability and EGFP expression in Jurkat cells. Cells were treated with KU55933 at different concentrations for 24 hours post-pulsing. **(A)** Cell viability; **(B)** eTE; **(C)** Expression Level; **(D)** Effective Expression Level. Pulsing condition: 650 V/0.2 cm, 400 μs, 1 pulse. Error bars: SEM; * P < 0.05, Student's t-test. N = 4.

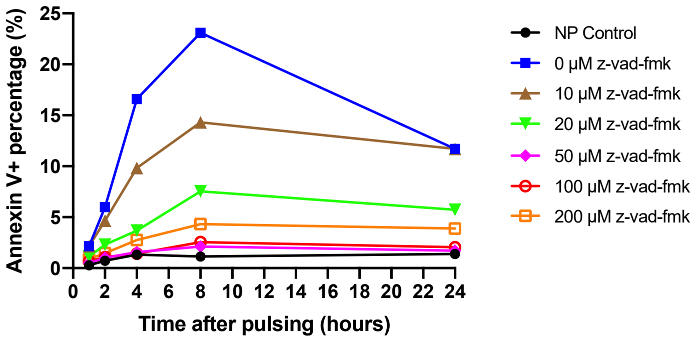

Figure S3. Effects of z-vad-fmk treatment on apoptosis of Jurkat cells. The percent of apoptotic cells was quantified with the Annexin V apoptosis assay. The experimental condition was similar to that in Figure 1, except that the cells were collected at 1, 2, 4, 8, and 24 hours post pulsing by centrifugation. The peak of apoptosis occurred at ~8 hours. Pulsing condition: 650 V/0.2 cm, 400 μs, 1 pulse. Each symbol represents data from a single experiment.

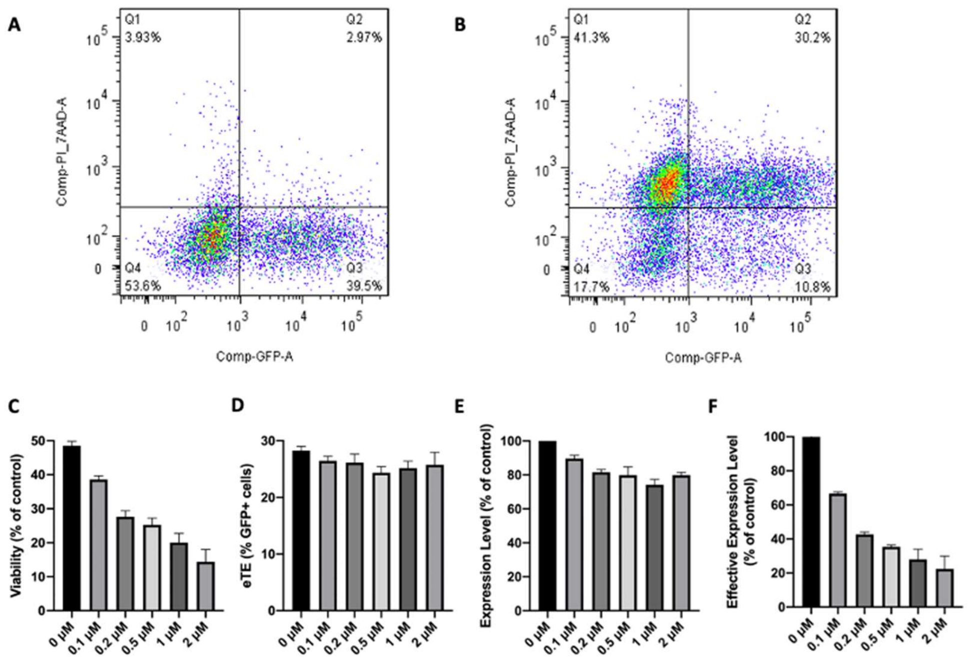

Figure S4. Effects of z-vad-fmk treatment on viability of human primary T cells. The experimental condition was similar to that in Figure 1, except that human primary T cells instead of Jurkat cells were used in the experiment. **(A)** Flow cytometry analysis of human T cells at 24 hours after pulsing without z-vad-fmk treatment; **(B)** flow cytometry analysis of human T cells at 24 hours after pulsing with 2 μM z-vad-fmk treatment. More than 70% of treated T cells became PI positive. **(C)** Cell viability; **(D)** eTE; **(E)** Expression Level; **(F)** Effective Expression Level for samples treated with z-vad-fmk at different concentrations. Pulsing condition: 650 V/0.2 cm, 400 μs, 1 pulse. N = 3.

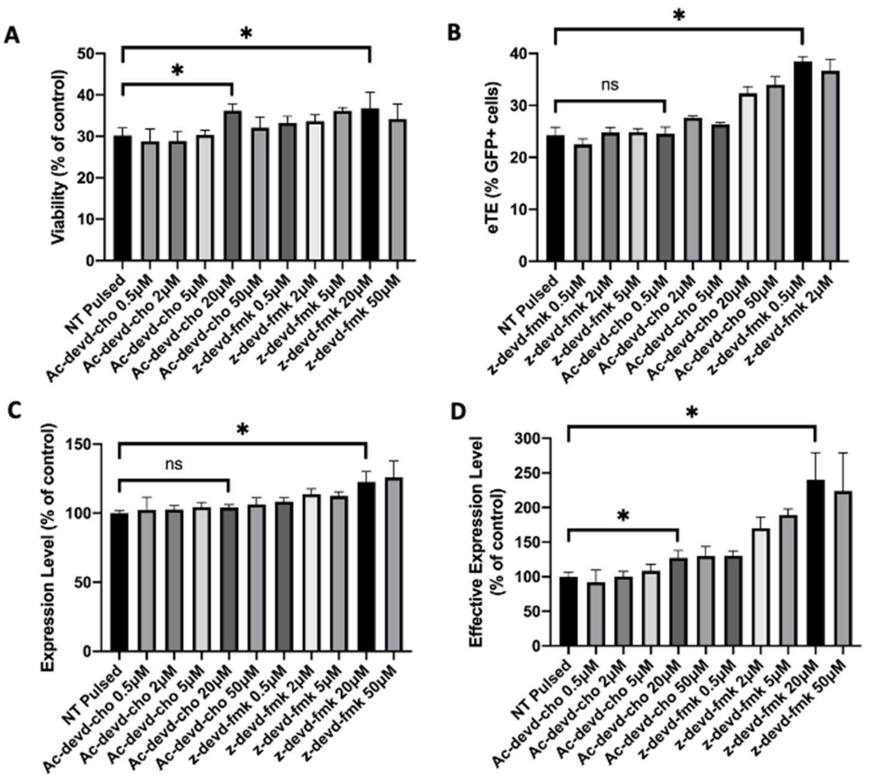

Figure S5. Effects of two caspase inhibitors (z-devd-fmk and Ac-devd-cho) on cell viability and EGFP expression in human primary T cells. The experimental condition was similar to that in Figure 1, except that human primary T cells instead of Jurkat cells were used in the experiment, and that z-devd-fmk and Ac-devd-cho, instead of z-vad-fmk, were used to inhibit cell apoptosis. **(A)** Cell viability; **(B)** eTE; **(C)** Expression Level; **(D)** Effective Expression Level. Pulsing condition: 650 V/0.2 cm, 400 µs, 1 pulse. ns: non-significant. Error bars: SEM; * $P < 0.05$, Student's t-test. N = 3.

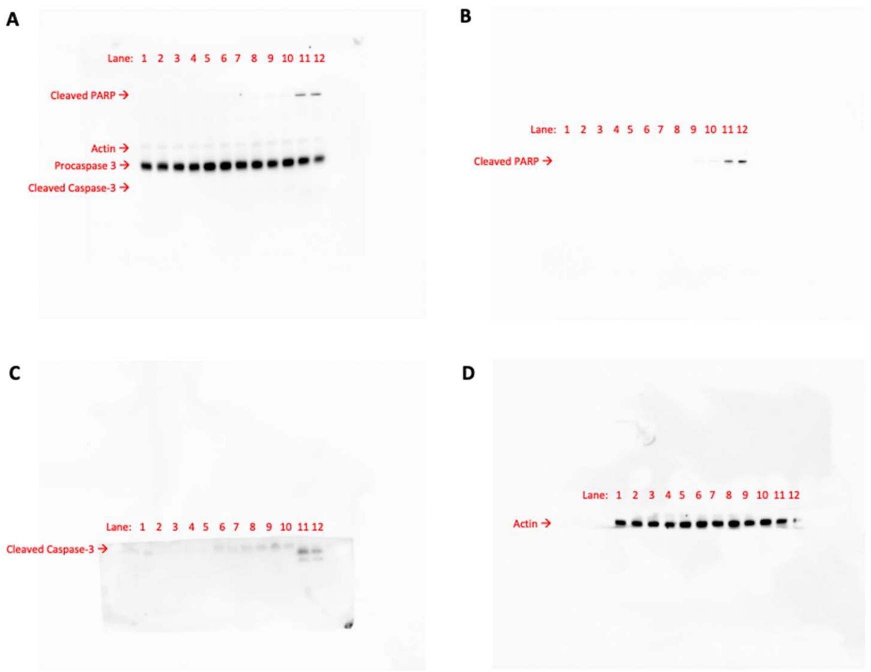

Figure S6. Original Western blot images used to generate the NP control panel in Figure 3A. Jurkat cells were treated with z-vad-fmk at different concentrations for 8 hours post pulsing. Western blot membrane was first imaged to show all areas **(A)**, then cut horizontally into three parts **(B-D)** to achieve optimal exposure time for imaging of different protein bands. **(A)** image of the whole membrane; **(B)** membrane portion containing the cleaved PARP bands; **(C)** membrane portion containing the cleaved caspase 3 bands; **(D)** membrane portion containing the actin bands. Pulsing condition: 650 V/0.2 cm, 400 µs, 1 pulse. Lane 1: 0 µM; Lane 2: 10 µM; Lane 3: 20 µM; Lane 4: 50 µM; Lane 5: 100 µM. Lane 6-10: repeats of lane 1-5; Lane 11&12: pulsed samples (positive controls).

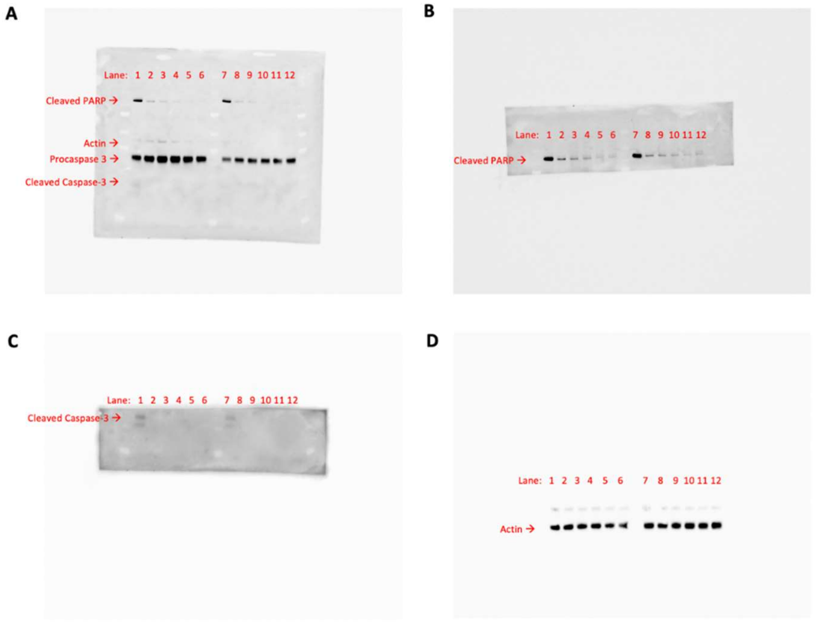

Figure S7. Original Western blot images used to generate the two panels for pulsed groups in Figure 3A. Jurkat cells were treated with z-vad-fmk at different concentrations for 8 hours post pulsing. Western blot membrane was first imaged show all areas **(A)**, then cut horizontally into three **parts (B-D)** to achieve optimal exposure time for imaging of different protein bands. **(A)** image of the whole

membrane; **(B)** membrane portion containing the cleaved PARP bands; **(C)** membrane portion containing the cleaved caspase 3 bands; **(D)** membrane portion containing the actin bands. Pulsing condition for Lane 1-6: 650 V/0.2 cm, 400 µs, 1 pulse. Lane 1: 0 µM; Lane 2: 10 µM; Lane 3: 20 µM; Lane 4: 50 µM; Lane 5: 100 µM. Lane 6: NP control (negative control); Pulsing condition for Lane 7-12: 550 V/0.2 cm, 300 µs, 2 pulses, 1 Hz. Lane 7: 0 µM; Lane 8: 10 µM; Lane 9: 20 µM; Lane 10: 50 µM; Lane 11: 100 µM. Lane 12: NP control (negative control).

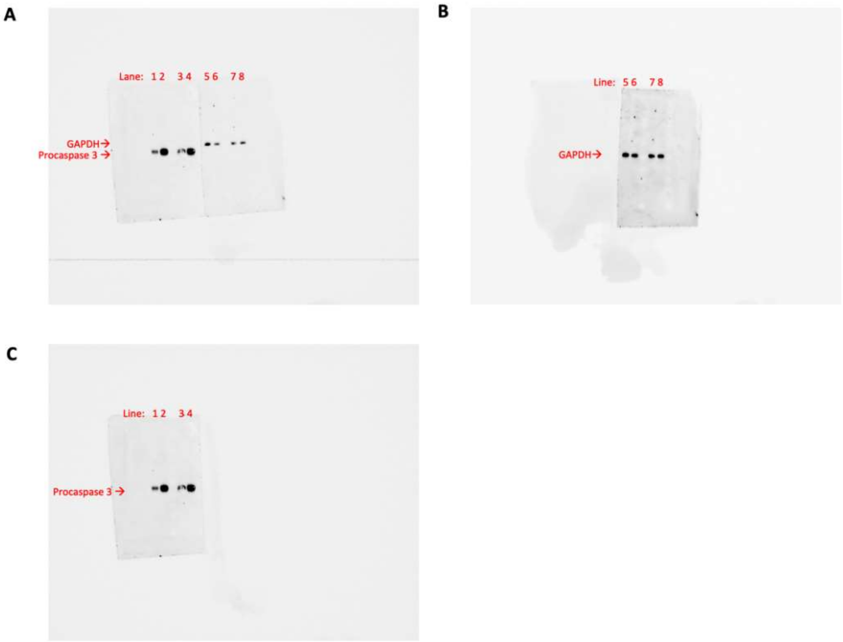

Figure S8. Original Western blot images used to generate Figure 4A. Jurkat cells were treated with either non-targeting control siRNA (Ctrl siRNA) or procaspase 3 siRNA (CASP3 siRNA). **(A)** image of the whole membrane; **(B)** membrane portion containing the GAPDH bands; **(C)** membrane portion containing the procaspase 3 bands. Lane 1&5: cells treated with CASP3 siRNA (sample 1); Lane 2&6: cells treated with Ctrl siRNA (sample 1); Lane 3&7: cells treated with CASP3 siRNA (sample 2); Lane 4&8: cells treated with Ctrl siRNA (sample 2). During the primary antibody incubation, lane 1-4 were incubated with procaspase 3 antibody, and lane 5-8 were incubated with GAPDH antibody. The bands of two samples were similar to each other. Thus, only the bands of sample 1 were reported in Figure 4A.

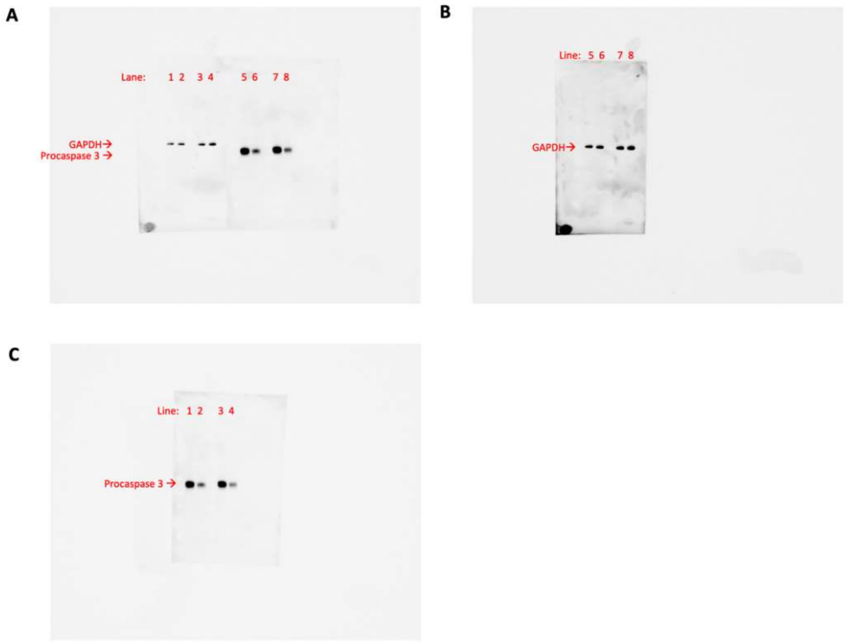

Figure S9. Original Western blot images used to generate Figure 6A. NIH/3T3 cells were treated with either non-targeting control siRNA (Ctrl siRNA) or procaspase 3 siRNA (CASP3 siRNA). **(A)** image of the whole membrane; **(B)** membrane portion containing the GAPDH bands; **(C)** membrane portion containing the procaspase 3 bands. Lane 1&5: cells treated with Ctrl siRNA (sample 1); Lane 2&6: cells treated with CASP3 siRNA (sample 1); Lane 3&7: cells treated with Ctrl siRNA (sample 2); Lane 4&8: cells treated with CASP3 siRNA (sample 2). During the primary antibody incubation, lane 1-4 were incubated with procaspase 3 antibody, and lane 5-8 were incubated with GAPDH antibody. The bands of two samples were similar to each other. Thus, only the bands of sample 2 were reported in Figure 6A.

© 2020 by the authors. Licensee MDPI, Basel, Switzerland. This article is an open access article distributed under the terms and conditions of the Creative Commons Attribution (CC BY) license (http://creativecommons.org/licenses/by/4.0/).
